# A Fast Preprocessing Method for Micro-Expression Spotting via Perceptual Detection of Frozen Frames

**DOI:** 10.3390/jimaging7040068

**Published:** 2021-04-02

**Authors:** Vittoria Bruni, Domenico Vitulano

**Affiliations:** 1 Department of Basic and Applied Sciences for Engineering, “La Sapienza” Rome University, Via A. Scarpa 14-16, 00161 Rome, Italy; domenico.vitulano@uniroma1.it; 2 Istituto per le Applicazioni del Calcolo “M. Picone”, Consiglio Nazionale delle Ricerche, Via dei Taurini 19, 00185 Rome, Italy

**Keywords:** facial microexpressions, motion energy, preattentive vision, standard deviation, spatio-temporal filtering

## Abstract

This paper presents a preliminary study concerning a fast preprocessing method for facial microexpression (ME) spotting in video sequences. The rationale is to detect frames containing frozen expressions as a quick warning for the presence of MEs. In fact, those frames can either precede or follow (or both) MEs according to ME type and the subject’s reaction. To that end, inspired by the Adelson–Bergen motion energy model and the instinctive nature of the preattentive vision, global visual perception-based features were employed for the detection of frozen frames. Preliminary results achieved on both controlled and uncontrolled videos confirmed that the proposed method is able to correctly detect frozen frames and those revealing the presence of nearby MEs—independently of ME kind and facial region. This property can then contribute to speeding up and simplifying the ME spotting process, especially during long video acquisitions.

## 1. Introduction

In the last few years, non verbal communication has gained interest in different fields, such as forensic investigation, security devices, clinical diagnosis, marketing investigation and forecasting, etc. In this context, facial micro-expressions (MEs) play a fundamental role, as they reveal the actual internal emotional states and intentions of a subject [1,2]. Despite a fast development of both computer algorithms and video acquisition technologies, automatic ME detection is still a challenging goal [3,4]. In fact, MEs are characterized by very short durations, ranging from 1/25 to 1/5 of a second (recently relaxed to a maximum duration of 1/2 s) [5]. Since this short duration usually goes along with a very low intensity involving just a partial motion, MEs are very difficult to detect by human beings [6]. Nonetheless, they have distinctive peculiarities, as highlighted in their original definition [7]. Among them, three interesting ME features are that they:

- Are often misinterpreted or missed altogether;
- Occur in half a second or less;
- Unconsciously display concealed emotions,

where the term “conceal” can be read as a lack of action of the speaker who tries to hide emotions. This last point represents the cornerstone of the following well-known ME taxonomy [8,9]:

- *Simulated expression:* A microexpression is not accompanied by a genuine expression/emotion. This is the most commonly studied form of a microexpression because of its nature. It occurs when there is a brief flash of an expression, and then a return to a neutral state.
- *Neutralized expression:* A genuine expression is suppressed and the face remains neutral. This type of micro-expression is not observable due to the successful suppression of it by a subject.
- *Masked expression:* A genuine expression is completely masked by a falsified expression. Masked expressions are microexpressions that are intended to be hidden, either subconsciously or consciously.

Despite the existing taxonomies, it is somewhat difficult to capture MEs at first glance, especially in videos from uncontrolled environments. As Paul Ekman did in his pioneering works [10,11], this goal is often reached only after various video replays. In order to support experts in this field, different approaches have been proposed in the literature for automatic ME spotting and classification. However, their limited duration unavoidably implies the use of very accurate but time consuming methods, as a frame by frame analysis is necessary in the spotting process. This actually represents the main drawback of an accurate analysis, as, for example, 30 min of a standard video sequence with a rate equal to 25 fps (frames per second) would require the inspection of 30·60·25 = 45,000 frames. On the other hand, it is interesting to note that psychologists and detectives usually give attention to just a few video clips, i.e., those where something that is “not convincing” occurs, by automatically discarding the useless ones. In practice, they apply what it is intrinsically contained in the informal ME definition given in [9].

Based on this observation, this paper aims at contributing to quantitatively characterize what is “something not convincing”. In particular, it will be shown that such an “unnatural” component is composed of a few frozen frames that occur just before or immediately after a ME. The presence of those frozen frames proves that the speaker under examination is trying to hide something. In contrast to MEs, frozen frames have the advantage of allowing for automatic detection through a simplified version of the Adelson and Bergen model for motion perception [12]. This kind of approach is doubly advantageous. On the one hand, it is absolutely general and matches well with all types of MEs; on the other hand, it contributes to speeding up the ME spotting process, whose pipeline requires different and expensive procedures [2]. Specifically, frozen frame detection consists of a fast and automatic selection of those video temporal intervals (groups of frames—GOFs), where it is worth checking for the presence of MEs with more accurate but expensive methods. As a result, the proposed approach serves as an efficient preprocessing tool that shows some robustness to events that do not meet the hypothesis of static background, such as luminance changes and quantization. Thus it contributes to preventing eventual instabilities in the spotting pipeline that may interfere with the final result [2].

The remainder of the paper is as follows. The next section presents the motivations of the work. It includes a very brief description of the state-of-the-art methods for ME spotting and a short presentation of the perceptual model that inspired the work. Section 3 deals with the presentation of the proposed method for the automatic detection of frozen frames. Some experimental results performed on both controlled and “uncontrolled” videos are presented in Section 4, and the last section draws the conclusions.

## 2. Motivation of the Work

Facial microexpressions are defined as “very brief, subtle, and involuntary facial expressions which normally occur when a person either deliberately or unconsciously conceals his or her genuine emotions” [5,9,10]. They are then characterized by a peculiar temporal evolution that can mainly be summarized into five phases:

- Neutral phase: ME is still absent.
- Onset phase: ME starts.
- Apex phase: ME reaches its maximum expression.
- Offset phase: ME begins to dissipate.
- Neutral phase: ME disappears.

Even though this temporal chain describes the whole process, the classification introduced in the previous section clearly reveals that this chain may be altered by the subject’s consciousness in hiding its true emotions, making ME detection more difficult.

Automatic ME detection in a video usually consists of two phases: (i) spotting and (ii) recognition. The former is strictly related to ME temporal evolution and it consists of finding out those video frames containing an ME. Recognition implies ME classification according to the facial action unit system (FACS) [13], which encodes 44 facial deformations caused by muscle movements—each ME involves one or more action units. Both spotting and recognition require different sequential operations that can be time consuming, especially for preventing detection failure and misclassifications. In particular, spotting is required to be enough precise in order to have a successful classification. MEs spotting is composed of three main steps: preprocessing, feature description and ME detection—see [2] for a complete review. It requires accurate face preprocessing, especially in its first step. Several methods have been proposed in the literature. For example, frame by frame methods [14,15] are based on face features and frame classification, while temporal methods [8,16] track the amount of deformation incurred by a specific facial region during motion: high deformation in very few frames is expected for ME. The former are not robust to spontaneous MEs; the latter, even though suitable for detecting spontaneous MEs and for distinguishing between macro and microexpressions, are dependent on amplitude and temporal threshold settings and require processing distinct facial regions. Many papers directly exploit motion intensity in a short time period or facial features’ temporal differences [16,17,18]. Moreover, they depend on a predefined temporal window that limits their adaptivity to videos having different rates. Another class of methods focuses on specific temporal phases by looking for, for example, the apex frame through the characterization of geometric and/or appearance and/or saliency features of specific facial components [19,20,21,22,23]. A first attempt to define a perceptual fingerprint of ME has been done in [24] by looking at an ME as a perceptual discontinuity. Even though preliminary results are promising, the use of high-pass details suffers from some sensitivity to noise or local movements that can provide some false alarms, including eye blinking. An attempt to distinguish between MEs and eye movements has been presented in [25], where the phase variations between frames were analyzed through the Riesz pyramid.

Considering the amount of data to be processed, it would be then advantageous to have a method that selects those groups of frames (GOFs) where MEs probably occur, using arguments and tools that are independent of ME kinds and are implementable through few and fast operations. More specific and sophisticated methods for precise ME spotting can be then applied just to these selected GOFs.

To that end, inspired by some approaches that have been employed to solve some image processing problems [26,27,28,29,30], in this paper preattentive vision has been considered, with reference to human vision sensitiveness to motion. This choice was mainly motivated by the simple observation that ME perception seems to mainly be an instinctive and immediate visual mechanism. More precisely, ME is an unconscious response of the subject to an external source, and in turn, it is a stimulus that is unconsciously perceived by a third party. As a result, MEs have perceptual properties that are very general and independent of the specific ME type and context. These global perceptual properties have to be quantified through global information that conveys the “unconvincing” component, without additional details concerning the specific ME.

Hence, differently from most of the ME spotting literature, the aim of this paper is to rely on a general and global motion estimation that must be independent of the specific facial region, but dependent on the limited temporal duration of both the ME and the eye fixation. This goal becomes more relevant if one considers that ME motion shares some features with first order motion (luminance changes) but some others with second order motion (textures) [31,32]. As a result, MEs often do not represent significant temporal discontinuities in the temporal motion strength.

In order to better display the contributions of this paper, the next subsection gives a short description of the perceptual model that has been adopted in this paper.

### Perceptual Motion Estimation: The Adelson and Bergen Model

**Figure 1:**
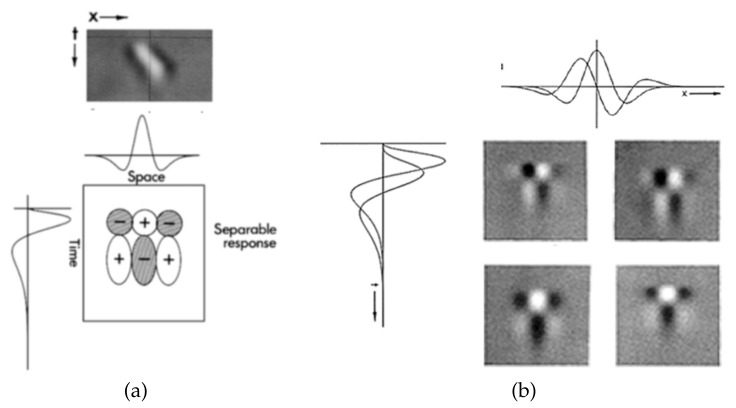
(**a**) Response of a spatio-temporal filter; (**b**) responses of a combination of couples of spatial and temporal filters having different supports.

As proved by several neurological studies [31,32,33], the human visual system is sensitive to motion even in the preattentive phase. The response of neurons that are sensitive to motion can be modeled as the impulse response of separable spatio-temporal filters. The combination of the single responses of specific spatio-temporal filters allows a good approximation of neurons’ sensitivity to motion direction (left/right). The idea is represented in Figure 1. A separable spatio-temporal filter is defined as

(1) h(x,y,t)=ρ(x,y)·ψ(t)

where the spatial filter ρ(x,y) is low-pass (sensitivity to object motion rather than single pixel motion) and the temporal filter ψ(t) is high-pass (sensitivity to temporal changes). The spatio-temporal filter provides the response in Figure 1a. The use of both spatial and temporal filters, having different supports, sets the sensitivity to motion velocity (slow or fast), as shown in Figure 1b.

Adelson and Bergen model [12] aims at quantifying the sensitivity to both velocity and motion direction in the case of low-level motion, such as legs or arms motion. To that end the two temporal high-pass filters are biphasic, i.e.,

$$\psi \left(t\right)={\left(kt\right)}^{n}\;e^{-kt^{2}}\left[\frac{1}{n!}-\frac{{\left(kt\right)}^{2}}{(n+2)!}\right],$$

with n,k fixed “a priori”. They are designed to detect both slow and fast motion. The low-pass spatial filters are selected so that one is odd and the other one is even—second and third-order derivatives of Gaussian functions are adopted in the original model, while Gabor functions have been used in successive modifications.

The combination of spatial and temporal filters defines a spatio-temporal filter that is able to replicate neurons’ sensitivity to motion. This represents a fundamental property for modeling motion perception. In fact, the four spatio-temporal filters can be further combined to provide directional (leftward and rightward) energies. The latter can be then subtracted to get the motion energy, conveying information concerning motion intensity, velocity and direction. Motion energy is a normalized quantity in the range [−1,1], and it is −1 for pure leftwards motion, +1 for pure rightwards motion and 0 whenever no directional energy is measurable. As it will be clearer in the following, the latter case will be of interest for our purposes.

## 3. Motion Perception and Frozen Frames

The proposed approach starts from the pioneering Adelson and Bergen model [12] and simplifies it in order to detect the absence of motion. The use of both spatial and temporal filtering is motivated by the following observations:

- *Space*: Human eye sensitivity is not at the pixel level; that is why pixel-based motion estimation, as optical flow, could result useless in this case as it could cause some additional but perceivable noise. Preattentive vision is characterized by fixation points [34] that are the centers of each observed region (foveated region) whose dimensions depend on the observation distance. This means that the farther an image point is from the fixation point, the more blurred it is perceived. As fixation points in the preattentive phase last 150–200 ms, while ME duration ranges from 100–166 ms to 500 ms, it immediately follows that the fastest ME reaches the limit of visual attention, while very few points, probably spatially correlated, are fixated during the longest ME lifetime [34]. As a result, an ME could not be in focus, with high probability, during the observation process, but it is equally perceived as a peripheral area of the field of view [32].
- *Time*: Visual perception is mainly based on contrast measures, i.e., the difference between the object of interest and its background; as a result, motion can be perceived as it causes a temporal contrast in the observed region. Temporal filtering is then necessary in order to quantify the temporal contrast, and then the temporal stimulus.

**Figure 2:**
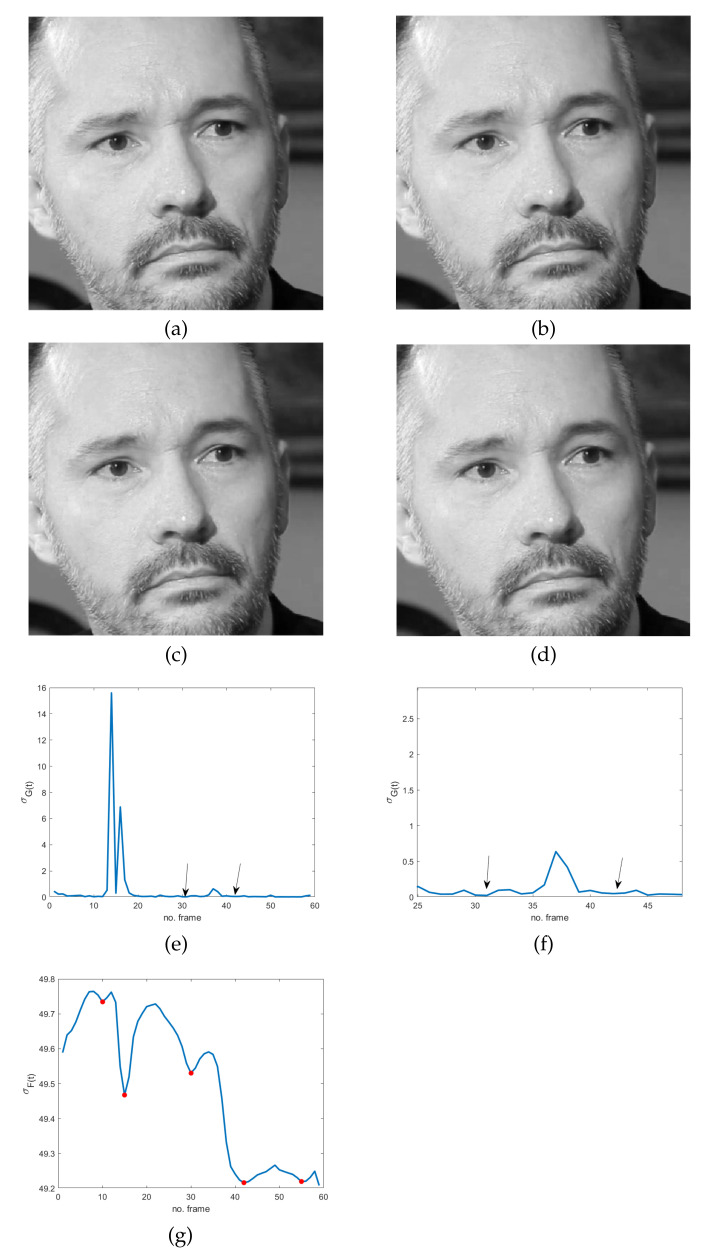
Four frames of the sequence cut 15.1 in the MEVIEW dataset [38]: (**a**) frozen frame before a microexpression (ME) ( surprise); (**b**) ME onset; (**c**) ME apex; (**d**) frozen frame after ME; (**e**) temporal global energy measured as the standard deviation of the spatio-temporal filtered sequence using the filter in Equation (1)—the arrows indicate frozen frames; (**f**) standard deviation restricted to the group of frames containing the ME; (**g**) standard deviation of the spatio-temporal filtered sequence using the filter l(x,y,t) in Equation (2) restricted to the group of frames containing a ME—markers correspond to local minima.

**Figure 3:**
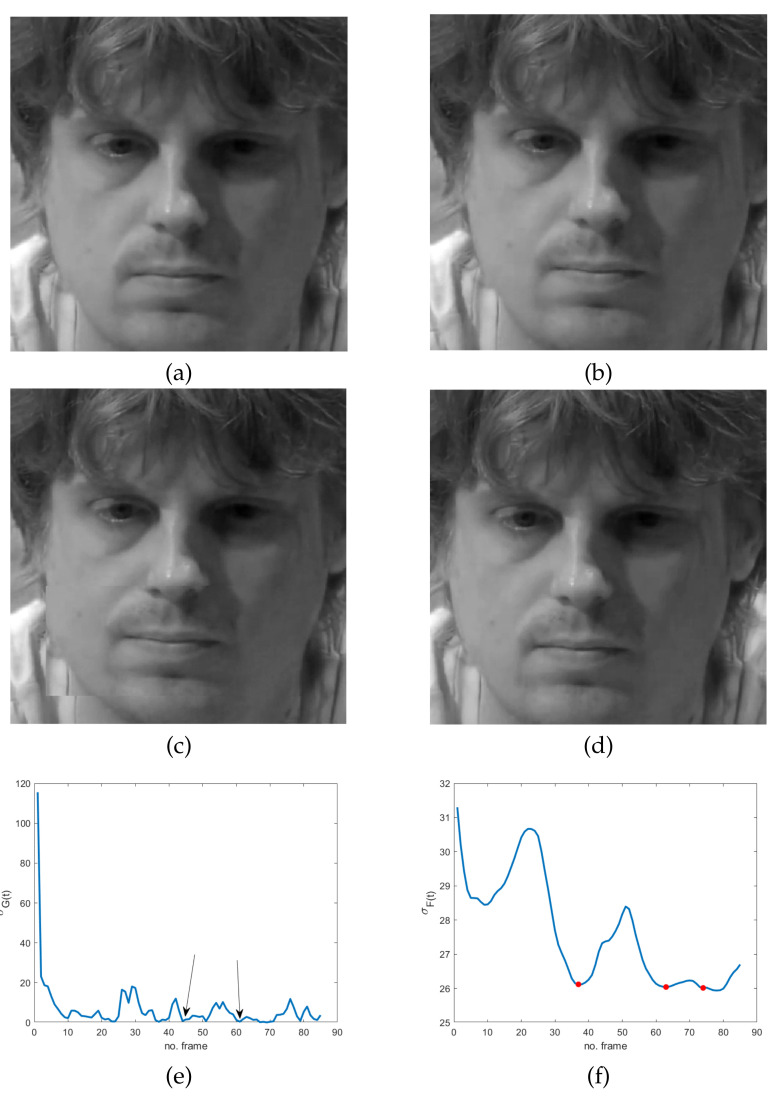
Four frames of the sequence cut 1.1 from the MEVIEW dataset [38]: (**a**) frozen frame before ME (contempt); (**b**) ME onset; (**c**) ME apex; (**d**) frozen frame after ME; (**e**) temporal global energy measured as the standard deviation of the spatio-temporal filtered sequence using the filter in Equation (1)—the arrows indicate frozen frames; (**f**) standard deviation of the spatio-temporal filtered sequence using the filter l(x,y,t) in Equation (2) restricted to the group of frames containing the ME—markers correspond to local minima.

**Figure 4:**
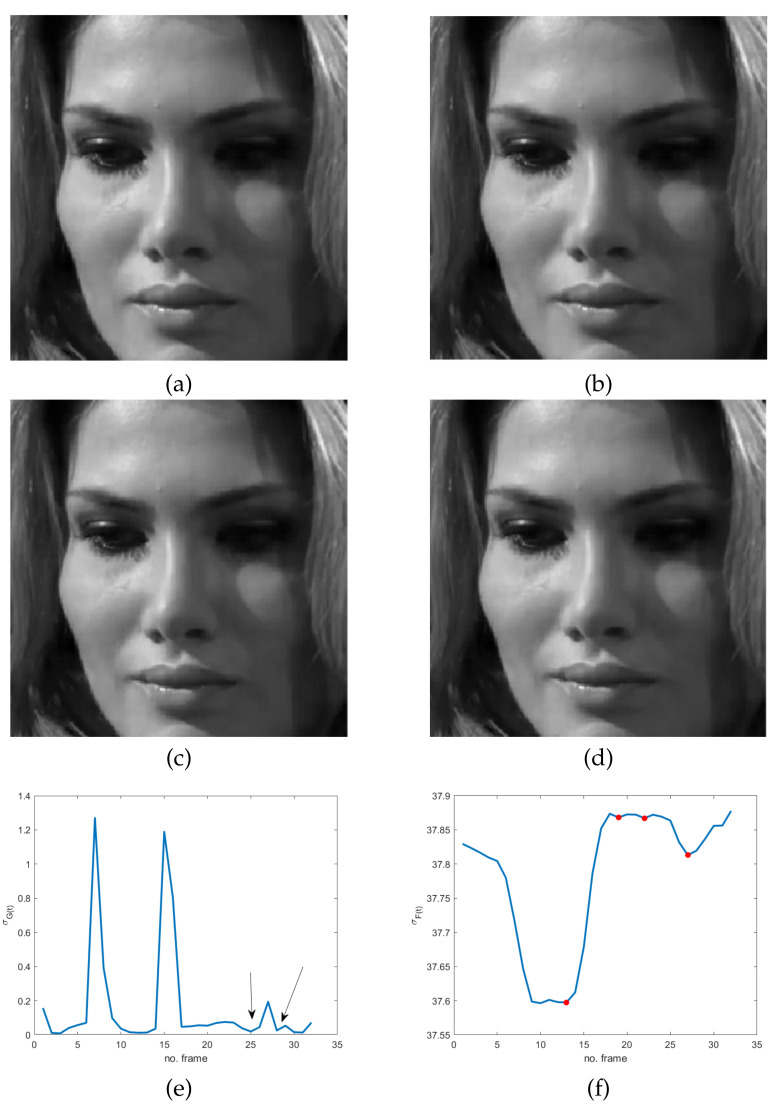
Four frames of the sequence cut 14.3 from the MEVIEW dataset [38]: (**a**) frozen frame before ME (happy); (**b**) ME onset; (**c**) ME apex; (**d**) frozen frame after ME; (**e**) temporal global energy measured as the standard deviation of the spatio-temporal filtered sequence using the filter in Equation (1)—the arrows indicate frozen frames; (**f**) standard deviation of the spatio-temporal filtered sequence using the filter l(x,y,t) in Equation (2) restricted to the group of frames containing the ME—markers correspond to local minima.

The simplified version of the model derives from the observation that very subtle and fast movements are of interest, independently of their direction. In addition, despite their unconscious nature, a sort of instinctive self-control mechanism is activated before or after MEs. In fact, the common attitude for hiding or suppressing emotions is to completely conceal oneself, causing frozen frames in the video sequence. This often happens in the offset phase but also just before the onset one. Even though it naturally occurs in controlled video acquisitions, like the ones in some datasets, including CASME II [35], SAMM [36] and SMIC [37], it could sound quite unreliable or at least much less evident or measurable in controlled videos than in ordinary conditions, such as the ones in the MEVIEW dataset [38]. As a matter of fact, it is not so. In order to give evidence of this statement, three sequences from the MEVIEW dataset have been considered (https://cmp.felk.cvut.cz/~cechj/ME/ (accessed on 1 April 2021); in all of them the subject maintains a concealed facial expression so that it is quite hard to detect pose differences in subsequent frames. In particular, in the first two sequences (Figure 2 and Figure 3), the subject intentionally tries to hide emotions by assuming a fixed posture that is completely concealed just before and immediately after the unconscious reaction (“surprise” and “contempt” respectively). In the third example (Figure 4), the concealed pose is assumed just after the unconscious reaction (“happiness”), with the apex expression lasting for different successive frames. This observation meets Ekman’s studies concerning MEs and automatically provides an additional ME feature that is independent of ME kind. More precisely, as frozen frames precede and/or follow any ME, they can act as a sort of early warning for the presence of MEs in a video sequence.

It is worth observing that this feature gives a practical advantage in the ME detection process. In fact, it is more convenient to detect the absence of motion rather than the fast motion of an ME. Among the different motivations, the most significant one is that ME causes motion that is neither completely of first order (luminance changes) nor of second order (textures) [31,32]. As a result, MEs often cannot be detected as isolated and significant temporal discontinuities in the motion intensity signal. In addition, the very short ME temporal duration limits one to the use of just one pair of spatio-temporal filters adopted in the Adelson and Bergen model, i.e., the one composed of an even low-pass filter in the space and a high-pass filter in the time having a very short support. This pair will be indicated as LH in the sequel and it defines a spatio-temporal filter like the one in Equation (1).

The use of frozen frames implies that a global absence of motion in the temporal sequence does involve the whole facial region; it means that we expect a minimum/zero in the energy of the response of the high-pass filter. Unfortunately, even though the latter is sensitive to very subtle and fast movements, it also has the same drawbacks of pixel-based motion estimators—i.e., artifacts are more visible than motion. As a result, the response of the spatio-temporal filter LH can be quite noisy, making ME detection troublesome and somewhat ambiguous. Specifically, local temporal minima can provide false alarms in the analyzed cases, as is evident in Figure 2e, Figure 3e and Figure 4e: several points, other than the ones corresponding to ME frozen frames in the energy signal, are close to zero. The energy signal is computed as the standard deviation of the spatio-temporal filtered video sequence using a filter as the one defined in Equation (1).

In the following, we prove that the high-pass temporal filter in the selected filter pair LH can be substituted for a suitable low-pass temporal filter in the spatio-temporal analysis, i.e.,

(2) l(x,y,t)=ρ(x,y)ϕ(t),

where ρ(x,y) is a spatial low-pass filter and ϕ(t) is a temporal low-pass filter—this filter pair will be denoted by LL in the sequel. In particular, it will be shown that the minima of the energy of the response of this new spatio-temporal filter LL include the zero points of the response of LH filters pair; in addition, due to its low frequency characteristics, the proposed spatio-temporal filter is more robust to the presence of noise or local artifacts in the video sequence.

In order to better characterize filters properties, it is worth reminding ourselves that in the preattentive phase, the human visual system takes only 13 images out of a second of continuous flow. As a result, the temporal filter is required to have a compact time support that depends on video sequence frame rate. By considering that a standard video sequence consists of 25/30 fps, the temporal sampling should be at least 1:2 in order to be consistent with the visual channel. As a result, in the simplified Adelson and Bergen model, the two separable filters (spatial and temporal) are both low-pass. Their supports are set according to both spatial and temporal resolution of the visual system, while the temporal energy is computed as the standard deviation of the spatio-temporal filtered sequence. The use of the standard deviation provides a global temporal variability measure of the video sequence, but it is also a crucial dispersion measure that is highly consistent with the vision process, especially with the preattentive phase [39].

Let us denote by

(3) G(x,y,t)=f*h

and

(4) F(x,y,t)=f*l

two spatio-temporal filtered versions of the original video sequence *f* that have been obtained by applying, respectively, LH (Equation (1)) and LL filter pairs (Equation (2)), and with

(5) $$\sigma_{U}\left(t\right)=\frac{1}{\sqrt{|\Omega_{U}|}}{∥U\left(x,y,t\right)-\mu_{U}\left(t\right)∥}_{2},$$

the spatial standard deviation of any function U(x,y,t) depending on both space (x,y) and time (*t*) variables, where $\mu_{U}$ is the corresponding mean value, while $|\Omega_{U}|$ is the dimension of the spatial domain. The next proposition proves that the minima of the standard deviation of *F* are strictly correlated to frozen frames, i.e., those characterized by lack of motion.

**Proposition** **1.** *Let G(x,y,t) and F(x,y,t) be two spatio-temporal filtered versions of the original video sequence f defined as in Equations (3) and (4), where*

(6) h(x,y,t)=ρ(x,y)ψ(t)andl(x,y,t)=ρ(x,y)ϕ(t),

*with ρ(x,y), a spatial low-pass filter; ψ(t), a temporal high-pass filter; and ϕ(t), a temporal low-pass filter such that $\psi \left(t\right)=\frac{d}{dt}\phi \left(t\right)$. Let $\sigma_{F}\left(t\right)$ and $\sigma_{G}\left(t\right)$ denote the spatial standard deviation—as defined in Equation (5)—of F(x,y,t) and G(x,y,t).*

*Then a local minimum for $\sigma_{F}\left(t\right)$ corresponds to a null value for $\sigma_{G}\left(t\right)$ (frozen frame). Conversely, null values of $\sigma_{G}\left(t\right)$ are realized in relation to local extrema of $\sigma_{F}\left(t\right)$.*

The proof is in Appendix A. What we observe here is that local minima of $\sigma_{F}\left(t\right)$ identify static scenes, i.e., frozen frames. As a result, this proposition provides a practical method for the detection of GOFs containing MEs. In fact, frozen frames delimiting MEs can be found among the local minima of $\sigma_{F}\left(t\right)$, which are easier to find with respect to $\sigma_{G}\left(t\right)$ zeros (see Figure 2, Figure 3 and Figure 4). In particular, as will be shown in the experimental results, frozen frames that occur before ME onset or after ME offset are identified by the absolute minima of $\sigma_{F}\left(t\right)$, when the latter is computed in relation to stationary scenes—see also Figure 2g, Figure 3f and Figure 4f.

### 3.1. The Algorithm

The proposed frozen frames detection algorithm can be summarized as follows.

- Partition the video sequence into stationary scenes $f_{k}\left(x,y,t\right),\;k=1,2,...$
- For each sequence $f_{k}\left(x,y,t\right)$:
  1. Detect a region Ω containing the face of interest in the whole subsequence $f_{k}$
  2. Set $f\left(x,y,t\right)=f_{k}\left(x,y,t\right)\left|{}_{\Omega }\right.$ and define F(x,y,t), as in Equation (4), for each *t* by applying the spatio-temporal filter *l* as defined in Equation (2) and in Proposition 1.
  3. Compute the spatial standard deviation $\sigma_{F}\left(t\right)$ of F(x,y,t) at each time *t* using Equation (5) by setting U=F
  4. Compute the local minima of $\sigma_{F}\left(t\right)$ and let ${\left\{t_{k}\right\}}_{0\leq k\leq M}$ denote their location.
  5. Remove eventual instabilities as follows:
    - Let $I={\left\{t_{k_{j}}\right\}}_{j}$ be a subset of adjacent local minima such that $|t_{k_{j-1}}-t_{k_{j}}\left|\leq d\;\mathrm{and}\;\right|\sigma_{F}\left(t_{k_{j-1}}\right)-\sigma_{F}\left(t_{k_{j}}\right)|\leq \tau$, with *d* and τ predefined values.
    - Remove them from the list of local minima
    - Set $t_{\hat{k}}$ equal to the mid-point of the set *I*.
    - Add $t_{\hat{k}}$ to the list of local minima.
    - Denote with ${\left\{{\bar{t}}_{k}\right\}}_{k}$ the modified sequence of local minima.
  6. Sort the local minima in ${\left\{{\bar{t}}_{k}\right\}}_{k}$ in ascending order (with respect to their value) and select the first *K* ones.
  7. Select a GOF around each selected local minimum location.
  8. Apply a suitable spotting algorithm to each GOF.

Details concerning steps 5–7 are provided in the next section.

## 4. Results and Discussion

The results presented in this section aim at giving evidence of the potential of the adopted visual perception based model for MEs in carrying out a fast but effective selection of those frames containing what has been defined as “something not convincing” in the Introduction. To that end, the proposed preprocessing method has been tested on different video sequences contained in publicly available and annotated spontaneous ME databases in order to have various ME types and subjects and different backgrounds and scenes. Even though the proposed method aims at working in the case of “in the wild” video sequences, some results concerning the case of datasets composed of acquisitions made under controlled conditions will be presented in order to assess the consistency and the reliability of its responses. In particular, results achieved on CASME II dataset [35] are presented in this section. CASME II is one of the largest and widely used databases; it is an improved version of CASME dataset and it contains a quite comprehensive representation of spontaneous MEs. It consists of about 255 videos that were recorded using high frame-rate cameras (200 fps). As videos were recorded under controlled conditions, several kinds of artifacts are missing, so more stable results are expected. With regard to more realistic scenarios, the MEVIEW dataset [38] has been considered. This dataset collects mostly poker game videos downloaded from YouTube. The peculiarity of this dataset consists in the fact that poker players often try to conceal or hide their true emotions—as a consequence, the corresponding videos contain several MEs. The dataset is composed of 31 videos having different subjects; videos were acquired at 25 fps; ME onset and offset frames are also provided.

A spatial Gaussian low-pass filter has been used for the spatial filter ρ in Equation (2), having standard deviation equal to 9. This dimension is consistent with a viewing distance that resembles the one used in real cases and which corresponds to approximately one degree of visual angle [34,40]. For the temporal low-pass filter, as mentioned in the previous section, the dimensions were set according to the sensitivity to motion as a continuous flow (i.e., 13 frames per second) and the frame rate of the video being analyzed. That is why it has been set as equal to 8 for the CASME II dataset and equal to 2 for MEVIEW. In order to eliminate some numerical instabilities that can create some local oscillations in $\sigma_{F}\left(t\right)$ and then false local minima, the mid-point of the minima that are measured in the instability regions is considered in the output list of local minima. A region is considered unstable if there exist subsequent local minima having comparable values (i.e., they differ for less than 10% of the energy range—τ=0.1 in step 5 of the algorithm) and whose distances are less than a predefined value *d* that depends on the frame rate of the analyzed video.

To quantitatively evaluate the results, two different tests have been run. The first test aims at giving empirical evidence of the concealing property of MEs and at showing that the proposed simple global measure ($\sigma_{F}\left(t\right)$) is able to identify them as local minima. The second test aims at confirming that the proposed temporal index allows us to select time intervals involving ME with a certain degree of reliability and with a simple and fast algorithm. This allows for spending the computational effort of a spotting algorithm only for a reduced temporal interval.

With regard to the first test, the CASME II dataset has been considered. In order to assess if the proposed method is able to correctly detect frozen frames nearby ME, it is expected that at least a minimum occurs in $\sigma_{F}$ temporal signal close to ME, independently of ME kind. More precisely, since onset, apex and offset are available for all videos in CASME II, the local minimum of $\sigma_{F}$ occurring just before onset and the one occurring just after the offset have been considered. In agreement with [38], a frozen frame is considered to be correctly detected if there exists one minimum such that its distance from ME onset or offset is less than N/2, where *N* is the maximal expected length of a ME, i.e., N=64 for CASME II (200Hz). Using these settings and d=N/4 for the selection of instability regions, the proposed method provides a positive answer for 245 sequences out of 255. This result confirms that: *(i)* frozen frames can be good candidates as early warnings for the presence of MEs, and *(ii)* the proposed spatio-temporal filtering allows us to define a simple quantity that is able to well represent them (Proposition 1).

The second test is oriented toward providing a more practical procedure for extracting a limited number of GOFs to be used as input for a spotting algorithm, independently of ME kind and action units involved. To that end, we repeat the aforementioned analysis but we limit our counting to the first *K* minima having the lowest $\sigma_{F}$ values; we consider the percentage of correct assignments for increasing *K*. Results for CASME II dataset are in Table 1. As it can be observed, for each ME in the database, independently of the subject, there is at least a group of frozen frames that precedes or immediately follows it. These frames provide a local minimum in the standard deviation signal $\sigma_{F}\left(t\right)$, defined in Equation (4), and for many sequences it represents the absolute minimum. In particular, frozen frames corresponding to MEs can be found among the first five minima for a high percentage of sequences (82%) and more than 50% among the first three. As a matter of fact, this percentage can increase if a more accurate removal of instabilities in the temporal signal $\sigma_{F}$ is performed. However, this is out of the scope of the preliminary study made in this paper.

**Figure 5:**
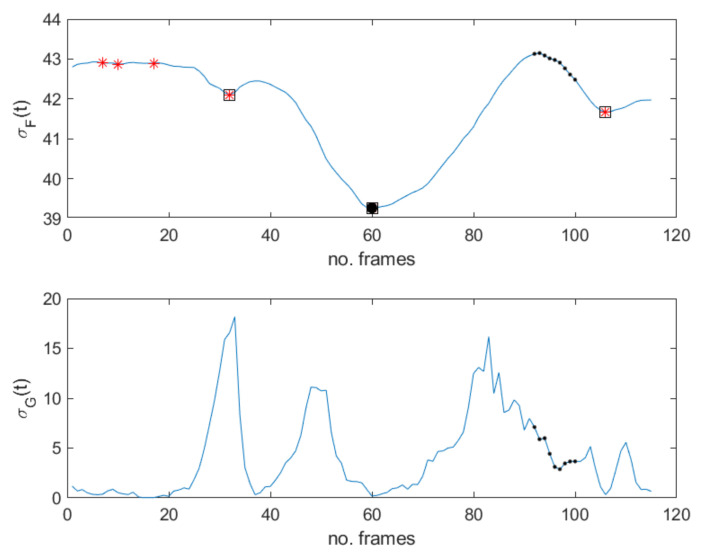
Sequence 2.1 in MEVIEW database. (**Top**) $\sigma_{F}\left(t\right)$ signal. Star makers correspond to local minima; square markers are the three selected minima—the absolute one is emphasized. Dots correspond to frames involving MEs. (**Bottom**) $\sigma_{G}\left(t\right)$ signal. Dots correspond to frames involving MEs. They do not correspond to evident impulsive peaks.

Based on these results, the same test has been applied to the MEVIEW dataset and the first three smallest minima of $\sigma_{F}$ have been considered; *N* has been set equal to 8 as 25 Hz as the rate for this dataset. In this case, we observed that the percentage of correct assignments is 90.32%. Some results are in Table 2. In particular, the frame numbers of the first three local minima are provided for several sequences; for each sequence, the local minimum that is closer to ME is in bold, while the absolute minimum is underlined. As it can be observed, the absolute minimum is nearby the ME of interest for the 40% of the sequences analyzed, even though the latter have not been acquired in controlled conditions—subjects moved, as did the camera position. Furthermore, frozen frames characterizing MEs correspond to one of the first three minima of $\sigma_{F}$, even when the subject moves, as is the case represented in Figure 5. In this case, perfect match among subsequent frames is not guaranteed but any registration algorithm has been applied; nonetheless, while peaks and zero values in $\sigma_{G}$ are not able to characterize ME, minima in $\sigma_{F}$ can still do that. This observation further confirms the advantage provided by the proposed method even in terms of robustness. As shown in Figure 5, several peaks are present in $\sigma_{G}\left(t\right)$ profile, as defined in Equation (3), but they are not in relation to the main ME; on the other hand, nearly zero values in the same signal would provide a lot of false alarms. On the contrary, the use of $\sigma_{F}\left(t\right)$ produces a significantly reduced number of false alarms, resulting more robust than $\sigma_{G}\left(t\right)$.

Table 2 also gives evidence of the advantage in using the proposed algorithm as preprocessing in the spotting process. This advantage has been quantified, as the percentage of frames in a video sequence that cannot be discarded by the spotting algorithm. It is worth observing that the proposed method is not able to assess if the detected frozen frames occur before or after MEs. That is why a temporal interval centered at the detected minimum has to be considered for further analysis. The half amplitude of this interval is set according to the average MEs time and video resolution, i.e., 4–8 frames in case of common resolution in standard cameras (25 Hz) and 16–32 in case of high resolution cameras (200 Hz). The results for the MEVIEW dataset have been included in Table 2, where for each detected minimum a GOF composed of 10 frames has been considered. As it can be observed, the preliminary detection of frozen frames allows one to reduce the number of frames to be processed by a spotting algorithm of about 60% on average, ranging from 84% to 20%, resulting in a considerable computing time reduction and increased real time processing capabilities.

In order to study the dependence of the proposed method on the adopted parameters, the area under the ROC curve (AUC) for the CASME II dataset has been considered. The curve was constructed by computing the true positive rate (TPR), i.e., $TPR=\frac{TP}{TP+FN}$, and the false positive rate (FPR), i.e., $FPR=\frac{FP}{FP+TN}$, with TP, FP, TN and FN respectively being the numbers of true positive, false positive, true negative and false negative assignments. In particular, according to the Algorithm in Section 3.1, *K* minima in $\sigma_{F}$ signal have been selected, i.e., ${\left\{t_{k}\right\}}_{1\leq k\leq K}$; hence, a frame has been considered a true positive ME assignment if it belongs to an interval having size equal to *N* and centered at a minimum $t_{k}$ whose distance from ME onset or offset is less than N/2. ROC curve has been then constructed by increasing *K*. The AUC values corresponding to different parameters settings are provided in Table 3. In particular, the threshold τ and the spatio-temporal filters size have been considered in the evaluation studies. The other parameters, as mentioned above, have been set depending on the video resolution, in agreement with the state-of-the-art methods. As it can be observed, the value of τ can change moderately the final result, especially for small temporal filter lengths as temporal instabilities are more probable. For fixed τ, AUC moderately changes according to temporal filter; however, to avoid the suppression of ME contribution in the adopted global measure, the temporal filter length is required to not exceed the minimum length expected for a ME in a video with a specific frame rate. The default application mode of the proposed method, i.e., a filter size depending on some perceptual features and video resolution, represents, on average, a good default setting for a generic video sequence.

Finally, Table 4 compares the proposed method with two state-of-the-art spotting methods: HOOF (Histograms of Oriented Optical Flow) [41] and 3D HOG (Histogram Of Gradients) [42] based ME spotting methods. The latter employ features that are commonly used to describe micro-expressions but that can be computationally demanding. As it can be observed, even though the proposed method is based on a global frame feature and does not apply any preprocessing method oriented to detect specific facial ROIs, it is able to provide comparable and even better results than the two competing methods.

## 5. Conclusions

In this paper a first attempt to accelerate the micro-expression spotting process has been presented. The method aims at reducing the temporal length of a video by discarding those frames that do not contain a facial ME with high probability. To that end, a fast and global method has been proposed that is based on the relation between some ME features and the human visual perception. In particular, the sensitivity to motion has been considered and a simplified and modified version of the motion energy model has been defined. Differently from existing methods and models, the proposed one looks at frozen frames rather than those revealing impulsive motion. In fact, frozen frames are strictly related to concealed poses that are assumed just before or immediately after MEs. Preliminary experimental results on a dataset with uncontrolled conditions showed that concealed frames actually characterize MEs, independently of the subject and ME kind. In addition, the modified motion energy model results are somewhat robust to background motion. Finally, the algorithm for the detection of frozen frames is simple and fast, so is computationally advantageous.

The achieved promising results motivate future research concerning this topic. In particular, global scene motion could be considered in order to further characterize the selection of interesting points without ambiguities. Moreover, the combination of the proposed features and the ones gathered from high-pass motion information would contribute to making the early warning reliable. Finally, the visual properties of facial MEs are worth further investigation.

## Figures and Tables

**Table 1:** CASME II dataset. Number of correspondences between minima of the temporal quantity $\sigma_{F}\left(t\right)$ and frozen frames’ nearby MEs. The correspondences were searched for among the first *K* local minima of $\sigma_{F}$ having the smallest values. The percentage of correct assignments with respect to the total number of analyzed video is also provided.

No. of Minima	1	2	3	5	8	10
no. of correct						
assignments	79	127	169	210	226	228
% of correct						
assignments	30.98	49.80	66.27	82.35	88.63	89.41

**Table 2:** Frozen frames detection results achieved on the MEVIEW dataset [38]. For each video subsequence (first column), the frame interval including the ME is provided (second column), along with the location of the first three absolute minima, if any, of $\sigma_{F}\left(t\right)$ (third column): the closest location to the ME interval is in bold, while the location where the absolute minimum is realized has been underlined. The last column contains the percentage of frames that need to be analyzed with a more precise spotting algorithm: for each minimum, 10 frames have been considered (5 before and 5 after). The length of the reference video-cut is indicated in the brackets.

No.	ME	$\sigma_{F}\left(t\right)$ Minima	Percentage
Sequence	Interval	Locations	(%)
1.1	[49 62]	37 **63** 74	33% (89)
2.1	[92 100]	32 60 **106**	25% (120)
3.1	[82 90]	68 **80** 111	22% (138)
5.2	[75 81]	54**72** 85	17% (174)
6.1	[15 26]	**12** 30 51	50% (60)
7.1	[52 60]	39 42 **48**	43% (69)
7.3	[93 98]	17 56 **89**	30% (101)
7.5	[54 70]	31 34 **50**	39% (77)
7.6	[59 76]	47 **56** 83	22% (136)
7.8	[81 90]	53 59 **80**	27% (112)
7.9	[76 87]	45 **88** 90	30% (100)
8.2	[19 34]	**18** 33 42	27% (109)
9.1	[88 96]	76 83 **88**	55% (55)
10.1	[13 27]	**10** 52 62	73% (41)
10.2	[81 93]	10 62 **80**	16% (192)
11.2	[7 21]	11 **29** 35	79% (38)
11.3	[57 67]	32 **50**	27% (73)
11.4	[9 23]	21 **27** 33	48% (63)
11.5	[33 49]	20 38 **50**	48% (63)
13.1	[16 32]	**15** 38 50	47% (64)
13.2	[6 20]	**23**29 34	52% (58)
14.1	[35 41]	42	16% (64)
14.3	[21 26]	13 22 **27**	81% (37)
15.1	[36 41]	30 **42** 55	47% (64)
16.2	[45 52]	30 **46** 54	48% (62)

**Table 3:** CASME II dataset. AUC (area under the ROC curve) for the proposed method with different parameter settings. The size of the spatial filter ρ and of the temporal filter ϕ in Equation (1), along with the threshold τ used in the removal of instabilities (step 5 of the Algorithm), have been considered. (**Top**) For fixed ρ standard deviation (std), AUC is evaluated for variable ϕ size and τ; (**bottom**) for fixed ϕ size, AUC is evaluated for variable ρ standard deviation and τ. The best result is in bold.

ρ std	9								
ϕ size	4			8			16		
τ	0.01	0.1	0.2	0.01	0.1	0.2	0.01	0.1	0.2
**AUC** (%)	72.65	**74.28**	66.29	73.18	72.01	70.93	70.46	69.61	69.43
ϕ size	8								
ρ std	5			9			15		
τ	0.01	0.1	0.2	0.01	0.1	0.2	0.01	0.1	0.2
**AUC** (%)	73.65	72.55	71.17	73.18	72.01	70.93	74.21	72.62	70.97

**Table 4:** CASME II dataset. Comparisons between AUC (area under the ROC curve) values provided by the proposed method (with the best parameters setting), the 3D-HOG-based ME spotting method [42] and the HOOF-based ME spotting method [41]. The best result is in bold.

	HOOF [41]	3DHOG [42]	Proposed
AUC (%)	64.99	72.61	**74.28**

## Data Availability

The data generated during the study are available on request from the corresponding author.

## Abbreviations

The following abbreviations are used in this manuscript:

fps	frames per second
GOF	Group Of Frames
ME	Microexpression
MEs	Microexpressions

## Appendix A. Proof of Proposition

**Proof.** Without loss of generality, let us denote $\sigma_{F}\left(t\right)={∥F\left(x,y,t\right)-\mu_{F}∥}_{2}$ and $\sigma_{G}\left(t\right)={∥G\left(x,y,t\right)-\mu_{G}∥}_{2}$. By using the convolution product properties, since $\psi \left(t\right)=\frac{d}{dt}\phi \left(t\right)$, $\frac{d}{dt}F\left(x,y,t\right)=G\left(x,y,t\right),$ By keeping in mind that $\sigma_{F}\left(t\right)$, for fixed *t*, is a norm, from the triangle inequality, we have

(A1) $$\sigma_{F}\left(t+\Delta t\right)-\sigma_{F}\left(t\right)\leq \sigma_{F(t+\Delta t)-F(t)}\left(t\right)$$

where Δt≥0. For Δt small enough, we can apply Taylor expansion to both members of previous inequality, i.e.,

$$\frac{d}{dt}\sigma_{F}\left(t\right)\Delta t+\frac{\Delta t^{2}}{2}\frac{d^{2}}{dt^{2}}\sigma_{F}\left(\tilde{t}\right)\leq \sigma_{G\left(t\right)\Delta t+\frac{\Delta t^{2}}{2}\frac{d^{2}}{dt^{2}}G\left(\bar{t}\right)}\left(t\right)$$

with $\tilde{t}\in \left[t,t+\Delta t\right],\bar{t}\in \left[t,t+\Delta t\right]$, that is equivalent to

(A2) $$\frac{d}{dt}\sigma_{F}\left(t\right)+\frac{\Delta t}{2}\frac{d^{2}}{dt^{2}}\sigma_{F}\left(\tilde{t}\right)\leq \sigma_{G\left(t\right)+\frac{\Delta t}{2}\frac{d^{2}}{dt^{2}}G\left(\bar{t}\right)}\left(t\right),$$

where the norm scaling property has been applied to the second member using Δt as scale value. Using similar arguments, it holds that

(A3) $$\sigma_{F}\left(t-\Delta t\right)-\sigma_{F}\left(t\right)\leq \sigma_{F(t-\Delta t)-F(t)}\left(t\right),$$

which is equivalent to

(A4) $$-\frac{d}{dt}\sigma_{F}\left(t\right)+\frac{\Delta t}{2}\frac{d^{2}}{dt^{2}}\sigma_{F}\left(\tilde{t}\right)\leq \sigma_{G\left(t\right)-\frac{\Delta t}{2}\frac{d^{2}}{dt^{2}}G\left(\bar{t}\right)}\left(t\right),$$

with $\tilde{\tilde{t}}\in \left[t-\Delta t,t\right],$ and $\bar{\bar{t}}\in \left[t-\Delta t,t\right]$ By using Equations (A2) and (A4) and letting Δt approach 0, it holds that

$$\left|\frac{d}{dt}\sigma_{F}\left(t\right)\right|\leq \sigma_{G(t)}.$$

As a result, in relation to frozen frames, i.e., $\sigma_{G(t)}=0$, $\frac{d}{dt}\sigma_{F}\left(t\right)$ must be zero—i.e., a relative extremum for it is expected. On the contrary, if $\hat{t}$ is a local minimum for $\sigma_{F}$, then the right-most sides of Equations (A1) and (A3) are positive and the equality holds. As a result,

$$0=\left|\frac{d}{dt}\sigma_{F}\left(t\right)\right|=\sigma_{G(t)};$$

i.e., a frozen frame is expected at time $\hat{t}$. □
